# Clinical validation of a graphical method for radiation therapy plan quality assessment

**DOI:** 10.1186/s13014-020-01507-5

**Published:** 2020-03-12

**Authors:** Tiago Ventura, Joana Dias, Leila Khouri, Eduardo Netto, André Soares, Brigida da Costa Ferreira, Humberto Rocha, Maria do Carmo Lopes

**Affiliations:** 1 Physics department, University of Aveiro, Aveiro, Portugal; 2 Medical Physics department, Portuguese Oncology Institute of Coimbra, Coimbra, Portugal; 3 Institute for Systems Engineering and Computers at Coimbra, Coimbra, Portugal; 4 Economy Faculty of University of Coimbra and Centre for Business and Economics Research, Coimbra, Portugal; 5 Radiotherapy department, Portuguese Oncology Institute of Coimbra, Coimbra, Portugal; 6 Radiotherapy department, Portuguese Oncology Institute of Lisbon, Lisbon, Portugal; 7 Radiotherapy department, Portuguese Oncology Institute of Porto, Porto, Portugal; 8 School Health Polytechnic of Porto, Porto, Portugal

**Keywords:** Decision-making, Plan quality assessment, Clinical validation

## Abstract

**Background:**

This work aims at clinically validating a graphical tool developed for treatment plan assessment, named SPIDERplan, by comparing the plan choices based on its scoring with the radiation oncologists (RO) clinical preferences.

**Methods:**

SPIDERplan validation was performed for nasopharynx pathology in two steps. In the first step, three ROs from three Portuguese radiotherapy departments were asked to blindly evaluate and rank the dose distributions of twenty pairs of treatment plans. For plan ranking, the best plan from each pair was selected. For plan evaluation, the qualitative classification of ‘Good’, ‘Admissible with minor deviations’ and ‘Not Admissible’ were assigned to each plan. In the second step, SPIDERplan was applied to the same twenty patient cases. The tool was configured for two sets of structures groups: the local clinical set and the groups of structures suggested in international guidelines for nasopharynx cancer. Group weights, quantifying the importance of each group and incorporated in SPIDERplan, were defined according to RO clinical preferences and determined automatically by applying a mixed linear programming model for implicit elicitation of preferences. Intra- and inter-rater ROs plan selection and evaluation were assessed using Brennan-Prediger kappa coefficient.

**Results:**

Two-thirds of the plans were qualitatively evaluated by the ROs as ‘Good’. Concerning intra- and inter-rater variabilities of plan selection, fair agreements were obtained for most of the ROs. For plan evaluation, substantial agreements were verified in most cases. The choice of the best plan made by SPIDERplan was identical for all sets of groups and, in most cases, agreed with RO plan selection. Differences between RO choice and SPIDERplan analysis only occurred in cases for which the score differences between the plans was very low. A score difference threshold of 0.005 was defined as the value below which two plans are considered of equivalent quality.

**Conclusion:**

Generally, SPIDERplan response successfully reproduced the ROs plan selection. SPIDERplan assessment performance can represent clinical preferences based either on manual or automatic group weight assignment. For nasopharynx cases, SPIDERplan was robust in terms of the definitions of structure groups, being able to support different configurations without losing accuracy.

## Background

The delivery of radiation therapy is based on a pre-calculated personalized dose plan optimized in a treatment planning system. A plan that simultaneously irradiates the target with the prescription dose and causes little or no damage to the organs-at-risk (OAR) and to the adjacent normal tissues is sought by the planner [1]. It is usually necessary to consider trade-offs between the dose delivered to the targets and the dose received by the normal tissues. So, each plan is a compromise solution between conflicting objectives. These compromises must generally be tackled by the human planner in an iterative manual trial-and-error process. Thus, plan optimization can be seen as a decision-making problem handled by a planner that attempts to simultaneously fulfil the dose prescription objectives and the tolerance dose criteria. As a result, the plan optimization phase is extremely dependent on the planner’s experience and on the complexity of the case and it cannot be guaranteed that the calculated plan or plans presented to the radiation oncologist (RO) are the best possible ones [2].

The clinical assessment of plan quality is typically done by verifying the fulfilment of the prescription dose in the target volume and the tolerance dose criteria for each OAR. The most common assessment methods used in the clinical routine are the visual inspection of the isodoses displayed on top of the computed tomography images and the evaluation of the dose-volume histograms (DVHs) and the corresponding dose statistics. The complexity of the plan and its possible impact on deliverability should also be considered. For instance, when comparing two plans with similar dose distribution, the one with lower number of beam incidences or/and number of monitor units should be selected, as the associated uncertainties tend to be lower. To yield a comprehensive appraisal of the quality of the 3D dose distribution, it is often necessary to take into account several dozens of parameters and that is not humanly possible [3]. If two or more of the best plans are to be compared, this task becomes even more demanding. As a result, plan selection is based on the information that the RO managed to hold or considered more relevant which may lead to unsystematic and/or subjective decisions.

As in many other medical fields, the RO decision about which plan should be elected for treatment is not only influenced by disease specific criteria (e.g. cancer stage, age, comorbidities or treatment toxicity) but also by the decision-maker individual characteristics (e.g. experience, emotions or degree of expertise) and by contextual factors (e.g. patient socioeconomic status, healthcare provider organization or political environment) [4]. Ideally, this complex decision-making framework should be supported by clinical reasoning methods able to efficiently combine targets, OARs and other normal tissues dosimetric data with the RO experience and clinical aims for a given pathology or the specific patient case. From the plan assessment point-of-view, treatment quality indexes describing the coverage [5] and conformity [6] of the target and/or the OARs sparing [7, 8] for radiosurgery treatments have been proposed some decades ago. With the generalization of inverse planning and multicriteria optimization techniques, other comprehensive figures of merit associating different types of dosimetric score combinations to assess the plan quality were also proposed [9–14]. However, the RO clinical preferences were just included in the scoring design of plan quality indexes proposed by Schultheiss and Orton [9] and by Jain et al. [10], through the application of statistical decision theory and decision analysis concepts, respectively.

Recently, a graphical method, named SPIDERplan, was developed to simultaneously assess and compare the quality of radiation therapy plans [15]. SPIDERplan considers the clinical aims associated with each of the structures of interest simultaneously weighting their relative importance.

The present work aims to assess whether it is possible to successfully relate SPIDERplan plan assessment with the RO clinical preferences. SPIDERplan was applied for plan selection considering nasopharynx cancer cases and the study design included two phases. In the first phase, pairs of plans were blindly and independently evaluated by three ROs. Afterwards, the configuration of SPIDERplan, in terms of groups weights, was automatically performed using a mixed linear programming model (MLPM) for preference elicitation. The plans that corresponded to the best SPIDERplan scores were then compared with the ROs plan choices. Intra- and inter-variability of the responses from the two phases were compared to conclude in what extent SPIDERplan was able to reproduce ROs choices. Finally, a threshold value for the score difference between competing plans, representing the value below which the plans can be considered as being of equivalent quality, was estimated.

## Methods

### Patient data

A sample of twenty nasopharynx cancer cases already optimized [16] was used for SPIDERplan clinical validation. Tumour stages included patients with stages I-IV (T1-T4, N1-N3a/3b and M0) that were delineated according to the Radiation Therapy Oncology Group and National Comprehensive Cancer Network guidelines. A simultaneous integrated boost prescription to be delivered in 33 fractions was assigned for all plans. The planning target volumes (PTVs), including tumour (PTV-T) or adenopathies (GTV-N) were prescribed with 70 Gy and the lymph nodes PTVs (PTV-N) with a dose range of 54.0 to 59.4 Gy (Table S1 of Supplementary material). The tolerance criteria of the spinal cord, the brainstem, the optics structures (chiasm, optical nerves, retina and lens), the pituitary gland, the ears, the parotids, the oral cavity, the temporomandibular joints, the mandible, the oesophagus, the larynx, the brain, the thyroid and the lungs, also contoured by the RO, were defined according to the nasopharynx clinical protocol of the Radiotherapy Department of the Portuguese Oncology Institute of Coimbra (Table S2 of Supplementary material).

### SPIDERplan description

SPIDERplan is a graphical method, developed by Ventura et al. [15], that uses a scoring approach to assess and compare the quality of radiation therapy treatment plans. It aims to address the dose prescription objectives, defined for the clinical case/pathology. SPIDERplan configuration is structured in two phases: the processing of the plan data and the assessment of the plan quality.

In the processing phase, targets and OARs are divided into groups according to the clinical protocol or the RO preferences. A pre-defined relative weight is attributed to each group and each structure, representing the clinical priorities during the plan evaluation. For each plan, a score based on the pre-defined planning objectives is calculated for each structure to express the fulfilment level of the corresponding planning goal.

In the plan assessment phase, a customised radar plot displays all the score information. Plan evaluation can be done by displaying all structures and groups information in a Structures Plan Diagram and in a Group Plan Diagram, respectively. Global plan score is determined as the weighted sum of the structures individual scores as:

1 $$ \mathrm{Global}\ \mathrm{plan}\ \mathrm{score}={\sum}_{\mathrm{i}}{\mathrm{w}}_{\mathrm{group}\left(\mathrm{i}\right)}{\sum}_{\mathrm{j}}{\mathrm{w}}_{\mathrm{struct}\left(\mathrm{j}\right)}{\mathrm{Score}}_{\mathrm{struct}\left(\mathrm{j}\right)} $$

where w_struct(j)_ and Score_struct(j)_ are the relative weight and the score of structure j, respectively, and w_group(i)_ the relative weight of group i. A partial group score based on the dose sparing of the structures that belong to that group is also calculated and represented in the Structures Group Diagram.

For the PTVs, the score was calculated according to a coverage criterion given by:

2 $$ {\mathrm{Score}}_{\mathrm{P}\mathrm{TV}}=\frac{{\mathrm{D}}_{\mathrm{TC},\mathrm{PTV}}}{{\mathrm{D}}_{\mathrm{P},\mathrm{PTV}}} $$

where D_TC,PTV_ corresponds to the tolerance criteria for the PTV (in this case the dose in 98% of the PTV that should be at least 95% of the prescribed dose) and D_P,PTV_ is the planned dose in the PTV. For the OARs, the score was set as:

3 $$ {\mathrm{Score}}_{\mathrm{OAR}}=\frac{{\mathrm{D}}_{\mathrm{P},\mathrm{OAR}}}{{\mathrm{D}}_{\mathrm{TC},\mathrm{OAR}}} $$

where D_P,OAR_ is the OAR planned dose and D_TC,OAR_ is the tolerance dose for each OAR.

A score of 1 is therefore expected when the dose of a given structure (target or OAR) is equal to the respective tolerance value. If either target coverage or OAR sparing are better than the goal set by the RO, the score will be less than one.

### SPIDERplan clinical validation

SPIDERplan clinical validation was performed in two-steps. In the first step, three ROs (RO1, RO2 and RO3), from three different national radiotherapy institutions, ranked and assessed the quality of the dose distributions of the selected cases. For each patient case, two plans (A and B), using coplanar optimized beam directions, were simultaneously presented to each RO. Based on the analysis of the dose distribution, the DVHs and the dose statistics (an example is provided for patient #3 in Fig. S1 of Supplementary material and Table S3 of Supplementary material, respectively), the ROs were asked to select the best plan of each of the 20 pairs of plans. If the plans were considered equivalent, both plans could be selected or rejected. For the evaluation of plan quality, each RO was asked to classify the plans as ‘Good’, ‘Admissible with minor deviations’ or ‘Not Admissible’. Four control cases were randomly selected and randomly introduced in the list of patients to evaluate the intra-rater variability of each RO. These control cases used the same plans of patient cases #1, #4, #6 and #9 and were displayed to the RO in a swapped position (Plan A replaced plan B and vice-versa).

In the second step, SPIDERplan evaluation was applied to the same 20-paired cases. Structures’ scores were determined for plan A and B according to eqs. 2 and 3 (Table S4 of Supplementary material). Two sets of structured groups were used to customize SPIDERplan response: a set of groups used by the local clinical protocol and a set of groups suggested by RTOG 0615 [17], named as CLIN and RTOG, respectively (Table 1). For the first set, SPIDERplan was successively applied using the CLIN group weights defined by the local RO (RO1) and the groups’ weights automatically generated by the MLPM method (CLIN_aut_), described in section 2.5. For the RTOG based groups, SPIDERplan evaluation just used the group weights defined by the MLPM method (RTOG_aut_).

**Table 1:** SPIDERplan group of structures defined locally according to RO aims (CLIN) and to RTOG guidelines (RTOG) [17]

CLIN		RTOG	
Groups	Structures	Groups	Structures
**PTV**	PTVs	**PTV**	PTVs
**Critical**	Brainstem	**Critical**	Retinas
	Spinal cord		Optical Nerves
			Chiasm
**Optics**	Lens		Brainstem
	Retinas		Spinal cord
	Optical Nerves		TMJ
	Chiasm		Mandible
**DigestOral**	Parotids	**Salivary**	Parotids
	Oral cavity		
	Larynx	**Other**	Brain
	Oesophagus		Lens
			Pituitary gland
**Bone**	Ears		Ears
	TMJ		Oral cavity
	Mandible		Larynx
			Oesophagus
**Other**	Brain		Thyroid
	Pituitary gland		Lungs
	Thyroid		
	Lungs		

*TMJ* Temporalmandibular joint

### Statistical analysis

The intra-rater and inter-rater variabilities of ROs for plan selection and evaluation were statistically assessed by the Brennan-Prediger kappa (K_B-P_) coefficient for nominal and ordinal variables, respectively [18]. The relative strength of the agreement is dependent on the K_B-P_ coefficient value and was classified using the scale proposed by Landis and Koch [19], where for K_B-*P*_ < 0.00 the agreement is ‘poor’, for 0.00 ≤ K_B-*P*_ ≤ 0.20 is ‘slight’, for 0.20 < K_B-*P*_ ≤ 0.40 is ‘fair’, for 0.40 < K_B-*P*_ ≤ 0.60 is ‘moderate’; for 0.60 < K_B-*P*_ ≤ 0.80 is ‘substantial and for 0.80 < K_B-P_ ≤ 1.00 the agreement is ‘almost perfect’.

### Automatic weight determination by mixed linear programming

When a decision-maker expresses his/her preferences by one out of two alternatives, the decision-maker is giving information regarding his/her preferences. It is possible to analyse these preferences, under a set of defined criteria, and to understand what is the importance that each one of the criterion has in the choice made. The importance of each criterion can be quantified by calculating a weight.

In this work, we have followed the methodology proposed by Srinivasan and Shocker [20]. Consider the multiattribute space defined by the different criteria that are taken into account by the decision-makers when making a choice. The decision-makers are the ROs. The multiattribute space dimension is equal to the number of different structure groups defined. Each attribute (criterion) is the corresponding structure group score. Each treatment plan is evaluated regarding the score of each one of the defined groups.

It is assumed that the ROs have a point in this multiattribute space that represents an ideal point: if a plan achieves, for each and all of the structures’ groups, the score defined by this ideal point, then they will be satisfied with the plan. Furthermore, it is assumed that ROs will prefer plans that are as close as possible to this ideal point. The problem of finding a vector of weights (one weight for each group) that is able to represent the ROs preferences can be represented by a mixed linear programming model, where the decision variables will be the weights. The objective will be to guarantee that the preferred plans are closer to the ideal point than the non-preferred plans.

The following notation is used:

#### Parameters

- *J* = {1,…,*n*} represents the set of plans that are going to be evaluated by the decision-maker
- *P* = {1,…,*t*} represents the *t* dimensions in which each of the plan is evaluated (each plan is evaluated considering each one of the groups so that *t* is equal to the number of groups considered)
- *Yj* ={*y*_*jp*_, *j* ∈ *J*, *p* ∈ *P*} represents the score of the *j*th plan for structure group *p*
- Ω = {(*j*, *k*), *j*, *k* ∈ *J*} represents the set of all ordered pairs (*j*,*k*) resulting from the comparison of plan *j* and plan *k* if *j* is preferred to *k*.

#### Decision variables

- *X* = {*x*_*p*_}, *p* ∈ *P* represents the ideal point to be determined
- *W* = {*w*_*p*_}, *p* ∈ *P* represents the weight of each one of the *t* dimensions (the weight that each group should have in the calculation of the global score).

It is possible to calculate the distance between each plan *j* ∈ *J* and the ideal point *X*. In this work we have chosen the Euclidean distance, meaning that:

4 $$ {d}_j=\sqrt{\sum_{p\in P}{\left({y}_{jp}-{x}_p\right)}^2},j\in J $$

If plan *j* was evaluated as being better than plan *k* then this should mean that *d*_*j*_ < *d*_*k*_, ∀ (*j*, *k*) ∈ Ω. The problem can then be described as: given *Y*_*j*_, ∀ *j* ∈ *J* and Ω, find *X* and *W* such that conditions (4) are violated as minimally as possible. It is thus necessary to define what is meant by “violating as minimally as possible”. In this work this has been defined as finding *X* and *W* such that the number of violations of eq. 4 is minimized.

Srinivasan and Shocker [20] showed that this problem can be represented by the following mixed linear programming model:

5 $$ \mathit{\operatorname{Minimize}}\ \sum \limits_{\left(j,k\right)\in \Omega}{\delta}_{jk} $$

subject to:

$$ \sum \limits_{p\in P}\left({y}_{kp}^2-{y}_{jp}^2\right){w}_p-2\sum \limits_{p\in P}\left({y}_{kp}-{y}_{jp}\right){v}_p+{\delta}_{jk}M\ge 0,\forall \left(j,k\right)\in \Omega $$

$$ \sum \limits_{\mathrm{p}\in P}\sum \limits_{\left(j,k\right)\in \Omega}\left({y}_{kp}^2-{y}_{jp}^2\right){w}_p-2\sum \limits_{p\in P}\sum \limits_{\left(j,k\right)\in \Omega}\left({y}_{kp}-{y}_{jp}\right){v}_p=1 $$

$$ {w}_p\ge 0,\forall p\in P $$

$$ {\delta}_{jk}\in \left\{0,1\right\},\forall \left(j,k\right)\in \Omega $$

where *M* represents an arbitrarily large positive number.

In the present work, Ω = {(*j*, *k*), *j*, *k* ∈ *J*} is built from the combined result of the evaluation made by the three different ROs. The objective was not to find *X* and *W* that would be RO dependent, but instead to find *X* and *W* capable of representing global preferences. This was achieved by applying a majority rule: (*j*, *k*) belongs to Ω if *j* was preferred to *k* by the majority of ROs.

## Results

### Plan selection and plan evaluation performed by the radiation oncologists

**Fig. 1:**
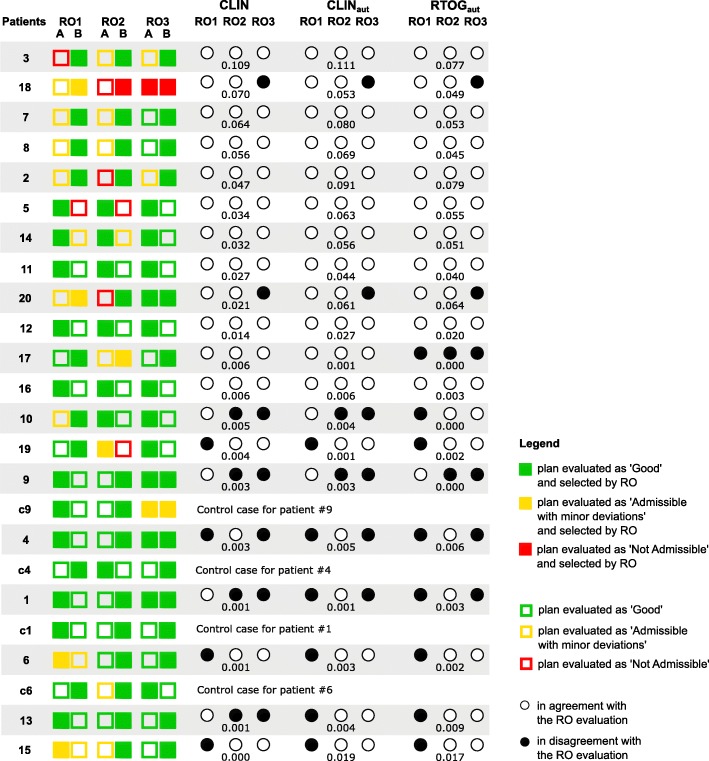
Plan selection and plan evaluation of ROs and agreement with SPIDERplan scoring. Legend: Fig. 1 - Results of the plan selection and evaluation of the selected nasopharynx cases by RO1, RO2 and RO3 (squares) and of the agreement and disagreement between SPIDERplan evaluation and the corresponding RO plan selection for CLIN, CLIN_aut_ and RTOG_aut_ group of structures (white and black circles, respectively). The difference between SPIDERplan global plan scores of plan A and B for the correspondent group of structures is shown immediately below. The patient cases were sorted in descending order of the CLIN set score difference. To facilitate the graphical comparison between the control cases and correspondent patient cases, the representation of plan A and B in the control cases was swapped

The results of plan selection and plan evaluation performed by the ROs are displayed in the first four columns of Fig. 1. For each comparison, the plan selected by the RO is represented by a filled square, the plans evaluated as ‘Good’ by a green square, the plans evaluated as ‘Admissible with minor deviations’ by a yellow square and the plans considered ‘Not Admissible’ by a red square. The control cases are represented in Fig. 1 below the correspondent patient case but were randomly presented to the ROs. More than two-thirds of the plans were evaluated as ‘Good’ by all ROs evaluations. Globally, all plans presented to the ROs, have high-quality dose distributions, but still 19% of the plans were evaluated as ‘Admissible with minor deviations’ and 8% as ‘Not Admissible’. For patients #4, #9, #18, #20 and the control case #c9 both plans A and B were selected by RO3, meaning that both plans were considered of equivalent quality. For patient #18, plans A and B were evaluated by RO2 and RO3 as ‘Not Admissible’ and for patient #20, plan A was differently evaluated by all ROs.

The intra- and inter-rater variabilities analyses were assessed through the calculation of K_B-P_ coefficients displayed in Table 2. The intra-rater variability was computed for each RO by comparing plan selection and plan evaluation of patients #1, #4, #6, #9 with the corresponding control cases. RO1 kept his plan selection for patients #1 and #9 and RO2 for patients #1, #6 and #9, which conducted to a fair agreement (K_B-*P*_ = 0.25). RO3 was the clinician with higher variability in plan selection, with a K_B-*P*_ = − 0.13, as he/she selected the same plan for patient #9 only.

**Table 2:** Intra-rater variability and inter-rater variability in plan selection and evaluation for each RO

	Intra-rater variability			Inter-rater variability		
	Radiation oncologist	Plan selection	Plan evaluation	Radiation oncologist	Plan selection	Plan evaluation
K_B-P_ coefficient	RO1	0.25 **Fair**	0.78 **Substantial**	All ROs	0.38 **Fair**	0.63 **Substantial**
	RO2	0.25 **Fair**	0.89 **Almost perfect**			
	RO3	−0.13 **Poor**	0.78 **Substantial**			

For plan evaluation, the intra-rater variability was unquestionably higher than for plan selection. When asked to grade plan quality, all ROs presented at least a substantial agreement between the first and the second evaluation (K_B-*P*_ = 0.78). RO1 and RO3 evaluated the quality of plans A and B as equally ‘Good’ for patients #1, #4 and #9 and for patients #1, #4 and #6, respectively. For RO2, the agreement was almost perfect (K_B-*P*_ = 0.89), as only for plan A of patient #6 the plan evaluation was not coincident.

For the quantification of inter-rater variability, the control cases were not considered. As in the previous analysis, the agreement between the ROs in plan selection (K_B-*P*_ = 0.38 - fair) was not as good as for plan evaluation (K_B-*P*_ = 0.63 - substantial). Only in 10/20 patient cases all ROs agreed in the selection of the best plan. While the agreement between RO2 and RO3 was high, the agreement between RO1 and RO2 and between RO1 and RO3 was about 50% (12/20 and 10/20, respectively). For plan evaluation, most of the plans (39/40) had two or more coincident RO ordinal assessments and more than one half (21/40) the same classification by all ROs. Again, it was between RO2 and RO3 that there was the higher number of plan evaluation agreements (29/40). For RO1, the number of plans with the same evaluation as RO2 and RO3 was almost equal (25/40 and 26/40, respectively).

### MLPM group weight determination

**Fig. 2:**
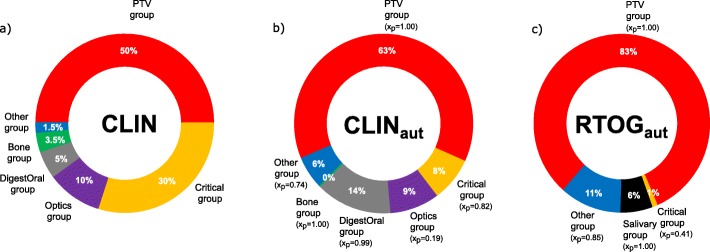
Group weights configured in SPIDERplan. Legend: Fig. 2 - Group weights for the CLIN, the CLIN_aut_ and the RTOG_aut_ sets. The x_p_ value represents the satisfaction point of the decision-maker

The group weights of CLIN, CLIN_aut_ and RTOG_aut_ group sets are shown in Fig. 2. The *CLIN* group weights (Fig. 2a) were defined according to the local clinical nasopharynx protocol and the CLIN_aut_ (Fig. 2b) and the RTOG_aut_ (Fig. 2c) were determined using the MPLM method. Values for vector *X* are also shown. Low values for *x*_*p*_ (namely less than one) mean that the ROs are, in reality, being more demanding with the corresponding group (finding plans satisfactory only if a low score is attained) than when greater *x*_*p*_ values are obtained. For the CLIN set, the PTV and the Critical groups received the higher weights (50 and 30%), while the Bone and the Other group the lowest weights. For the CLIN_aut_ set, the groups’ weights changed considerably. The PTV group presented the higher weight, but the Critical group weight was lower than those of the DigestOral and Optics groups. However, the *x*_*p*_ associated with the Critical group is less than one, showing that this is, in fact, an important structure group and the ROs will not, probably, be satisfied with plans that simply comply with the planning goal, expecting to see better organ sparing. For the Bone group the calculated weight value is 0% and the *x*_*p*_ is equal one, meaning that the dose received by the structures of this group will only have to comply with the prescribed value for the RO to be satisfied. For the RTOG_aut_ set, the PTV group achieved the highest weight, while the lowest was computed for the Critical group. This is the group with the lowest *x*_*p*_, certifying that the ROs value a low score from the structures of the group even if a relative low weight has been assigned to it.

### SPIDERplan evaluation

SPIDERplan global plans scores were computed for all patient cases using the groups and the weights from CLIN, CLIN_aut_ and RTOG_aut_ sets. Its response accuracy is graphically displayed in Fig. 1, where the agreement between the selection based on SPIDERplan global plan score and the clinical plan choice is represented by white circles and the disagreement by black circles. The patient cases are sorted by descent order of the score difference between plan A and B for the CLIN configuration. A complete agreement between SPIDERplan selection for all sets and all ROs was obtained in 9/20 of the patient cases and at least two agreements per set in 14/20 of the cases. It can be seen that the higher the global score difference between the two paired plans, the better the SPIDERplan results agree with the ROs choice and also the closer the agreement among the three ROs (e.g. patients #2, #3, #7 or #8). Globally, the agreement in plan selection between SPIDERplan and all ROs was high (> 45/60), resulting in an inter-rater variability of substantial to moderate agreement. The plan selection agreement between SPIDERplan and RO1 was higher for the CLIN set than for the remaining sets whose group weights were automatically determined by the MLPM method. On the contrary, the percentage of plan selection agreement between SPIDERplan and RO2 and RO3 was higher for the CLIN_aut_ and RTOG_aut_ sets than for CLIN where an inter-variability of almost perfect agreement was obtained. Nevertheless, the global percentage of agreement and the intra-rater variability (all ROs) was almost equal for all sets (45/60, 46/60 and 44/60). A total disagreement between SPIDERplan response and all ROs was just obtained for patient #17, when RTOG_aut_ set was used (three black circles). In this case the difference in plan quality between the two plans is so small (0.0008) that in fact it is irrelevant which is the plan selected for treatment. Thus, a threshold value for the score difference between two plans was defined. This threshold, estimated as 0.005, represents the value below which two plans are judged as dosimetrically equivalent. Considering now this threshold value, the agreement between SPIDERplan and RO plan selection increases from 45/60 to 55/60 cases. The plan choices made by SPIDERplan that fail this threshold value where #15 (CLIN_aut_ and RTOG_aut_) #18 and #20. For patients #18 and #20, RO3 could not make a choice between plans A and B, so no agreement could ever be found, anyway. For patient #15, RO1 was not in agreement with the other ROs and, as stated in section 2.5, his/her choice was thus not considered for the automatic determination of the group weights by MLPM method (the majority decision was considered).

## Discussion

SPIDERplan is a graphical plan assessment tool developed for supporting the clinical choice of the best plan for treatment delivery. The evaluation of the quality of the dose distribution is done by combining the graphical analysis provided by customised radar plots with a scoring index. Weighted groups of structures reflecting the RO clinical preferences for a given pathology or case must be defined and validated prior to starting using SPIDERplan in the clinical routine. In this study, SPIDERplan was clinically validated for the nasopharynx pathology by comparing the plan evaluation made by three ROs with the SPIDERplan score results.

Twenty nasopharynx cases with high-quality dose distributions were blindly evaluated and ranked by three ROs from different institutions. Four control cases were randomly selected from the list of these patient cases with the most similar plans and randomly presented to the ROs without their knowledge. The choice of the best treatment may be influenced by different factors (individual characteristics of the decision-maker and the patient, contextual factors, specific technical criteria), that can introduce some inter- and even intra-variabilities in the decision of the ROs. In this work, some of these factors were surpassed given the retrospective and anonymous character of the selected patient cases sample. The ROs assessed the plans following their own institutional protocol guidelines, using traditional treatment plan evaluation tools (dose distribution visualization, DVH and dose statistics analysis) and embedding into the final decision their personality and clinical experience. On average, just 3 hours were spent by each RO to complete the assessment of all the cases. On one hand, the continuous time slot dedicated to this task may have negatively influenced the consistency of his/her evaluation, as the repetition of cases assessment may have caused some inattention/fatigue to, at least, the last evaluated cases. Probably that was the reason for RO3 not having been able to select the best plan in patient cases #18 and #20 (the last ones) even with high score differences. On the other hand, it assured, in principle, the use of more consistent criteria during the process.

For plan selection, the intra-rater variability analyses presented lower K_B-P_ coefficients than the inter-rater variability analysis, meaning that the agreement between different ROs was better than between themselves. This low agreement may be a result of the high-quality of the dose distributions of the control cases and also the similarity of the plans in the control pairs. This choice of control plans avoided the perception, by the ROs, that control cases have been introduced because it was harder to acknowledge that they were comparing for the second time a pair of plans already considered. Of course, the reduced number of cases used for this intra-variability analysis statistically influenced the intra-rater agreement result. From the intra-rater and inter-rater variabilities analyses, it is also evident that the agreement between SPIDERplan and the ROs for plan evaluation was much higher than for plan selection. This finding has a direct correspondence with RO appraisal in the clinical routine as it is usually much easier for clinicians to agree upon the quality of the plans (saying if they are ‘Good’, ‘Admissible with minor deviation’ or ‘Not Admissible’) than it is to select the plan for treatment.

SPIDERplan response accuracy was tested using two sets of groups of structures and two methods to establish group weights. The composition and the weights of the CLIN set were defined according to the local nasopharynx protocol and the RO1 clinical preferences. SPIDERplan response reproduced ROs selection in 75% of the cases. It is interesting to note that the disagreement between SPIDERplan and ROs in plan selection was in-line with the inter-rater variability analysis between the three ROs corroborating the accuracy of SPIDERplan assessment. The small number of disagreements occurred for very low score differences between plans A and B. A threshold, in terms of score difference between plans, below which the choices were considered in agreement with ROs was thus defined. Values below this threshold reflected the difficulty showed by the ROs to choose the best plan when they were very similar. This threshold can be seen as a measure of the uncertainty associated with SPIDERplan plan assessment and also as a justification for the intra-rater and inter-rater variability of the ROs plan selection and evaluation.

The definition of groups of structures according to their clinical importance and the corresponding assignment of importance weights is a non-trivial task for ROs. In the daily routine ROs acceptability criteria and preferences are qualitatively incorporated in the process of selection and approval of the best plan and not based on a quantitative value reflecting the importance of each structure. Therefore, a MLPM method was applied to automatically determine the weights of each group (CLIN_aut_). An alternative group of structures was also defined following RTOG 0615 guidelines, and the respective weights calculated using the same automated method (RTOG_aut_). Compared to CLIN_aut_ and RTOG_aut_, SPIDERplan performance was similar to that of the CLIN set except for RO1 where the agreement for the two new sets of structures groups decreased. This is to be expected as the CLIN set was defined by the clinical protocol followed by RO1.

The group weights determined by the MLPM method considered the clinical choices of all ROs in plan selection. Indeed, the CLIN_aut_ set presented a somewhat different configuration from the CLIN set (Fig. 2). The unexpected low weight of the Critical group may have grounds on the automated method itself. The low score values (high sparing) and the associated low variability presented by this group give room to the MLPM algorithm to confer more importance to groups with scores with higher values and higher variability, such as the Optics, the DigestOral or the Salivary groups. Nevertheless, the lower *x*_*p*_ values showed that, although the importance of this group in the plan evaluation was not so high as initially thought, the ROs required that this group of structures presented higher levels of sparing to be satisfied.

SPIDERplan was configured for the nasopharynx pathology using a local group set and weights, a local group definition with weights automatically calculated, and also using a group definition based on international guidelines with automated group weights. The performance of SPIDERplan against the ROs choices, for all sets of group weights, was similar to the inter-rater variability obtained between the ROs clinical evaluations. The flexibility in plan evaluation and comparison provided by different group weights enables the possibility to adapt with confidence any of these SPIDERplan configuration options in the clinical practice. For other pathologies, any of these SPIDERplan configuration methods could be followed. It is possible to define the structure groups and weights resorting to the local RO team clinical protocols and preferences. Alternatively, it is possible to automatically elicit these weights through the analysis of the comparison of different plans using a pool of patient cases considering either groups locally defined or in accordance to international guidelines.

## Conclusions

In this work, the evaluation of SPIDERplan was successfully linked to the plan evaluation of three ROs from three Portuguese radiation therapy departments for the nasopharynx pathology using three different configuration methods. SPIDERplan plan evaluation agreed with most of the ROs assessments and presented an equivalent variability to that of the ROs choices. To handle decision uncertainty when the quality of the plans is very similar, a threshold value was determined for the score differences between the plans, below which the plans are considered of equivalent quality.

For the nasopharynx pathology, any of the configurations tested, i.e., based on local preferences or automatically determined from a pool of testing cases, can be used in SPIDERplan without loss of accuracy. For other pathologies, any of these configuration methods can/could be set before starting using SPIDERplan in clinical practice.

## Supplementary information

**Additional file 1:** Supplementary material. The supplementary material section prescription details of nasopharynx patients, tolerance dose criteria for PTVS and OAR, dose statistics, SPIDERplan scores and DVHs of patient #3.

## Data Availability

All data generated or analysed during this study are included in this published article [and its supplementary information files].

## Abbreviations

DVH: Dose-volume histogram
K_B-P_: Brennan-Prediger kappa coefficient
MLPM: Mixed linear programming model
OAR: Organs-at-risk
PTV: Planning target volume
RO: Radiation oncologist
RTOG: Radiation Therapy Oncology Group
TMJ: Temporalmandibular joint
