# Supplementary material for: Clinical validation of a graphical method for radiation therapy plan quality assessment
Source: Radiat Oncol. 2020 Mar 12;15:64. doi: 10.1186/s13014-020-01507-5 (PMC7068922; doi:10.1186/s13014-020-01507-5)
Supplement: Supplementary file 1 — Additional file 1: Supplementary material. The supplementary material section prescription details of nasopharynx patients, tolerance dose criteria for PTVS and OAR, dose statistics, SPIDERplan scores and DVHs of patient #3. [file 13014_2020_1507_MOESM1_ESM.pdf]

Supplementary material

Table S1 – Prescription details of nasopharynx patients, i.e. disease level, number of fractions, prescription doses and volumes of the lymph nodes PTVs (PTV-N1 and PTV-N2), tumour PTV (PTV-T) and adenopathies PTVs (GTV-N1, GTV-N2, GTV-N3).

Patient #	Disease level	Number fractions	PTV_T	GTV_N1	GTV_N2	GTV_N3	PTV_N1	PTV_N2
**1**	T2N3bM0	33	70.0 Gy (55.5 cc)	70.0 Gy (25.0 cc)	70.0 Gy (13.0 cc)		59.4 Gy (324.2 cc)	59.4 Gy (202.8 cc)
**2**	T2N3bM0	33	70.0 Gy (102.1 cc)	70.0 Gy (66.3 cc)	70.0 Gy (171.7 cc)	70.0 Gy (4.5 cc)	59.4 Gy (452.3 cc)	59.4 Gy (469.0 cc)
**3**	T4N0M0	33	70.0 Gy (100.4 cc)				59.4 Gy (352.7 cc)	59.4 Gy (172.7 cc)
**4**	T1N1M0	33	70.0 Gy (28.7 cc)	70.0 Gy (21.9 cc)			59.4 Gy (276.5 cc)	54.0 Gy (222.9 cc)
**5**	T2bN1M0	33	70.0 Gy (24.4 cc)	70.0 Gy (2.42 cc)			59.4 Gy (296.3 cc)	54.0 Gy (228.3 cc)
**6**	T2N3M0	33	70.0 Gy (37.1 cc)	70.0 Gy (187.5 cc)			59.4 Gy (523.2 cc)	54.0 Gy (221.2 cc)
**7**	T4N2M0	33	70.0 Gy (58.3 cc)	70.0 Gy (3.7 cc)	70.0 Gy (4.8 cc)		59.4 Gy (317.7 cc)	59.4 Gy (199.7 cc)
**8**	T4N2MX	33	70.0 Gy (49.7 cc)	70.0 Gy (41.2 cc)	70.0 Gy (5.2 cc)		59.4 Gy (364.9 cc)	59.4 Gy (245.3 cc)
**9**	T1N2M0	33	70.0 Gy (19.3 cc)	70.0 Gy (8.6 cc)			59.4 Gy (278.4 cc)	59.4 Gy (225.0 cc)
**10**	T2bN3aM0	33	70.0 Gy (20.6 cc)				59.4 Gy (219.3 cc)	59.4 Gy (154.5 cc)
**11**	T4N2M0	33	70.0 Gy (267.3 cc)				59.4 Gy (518.7 cc)	59.4 Gy (217.0 cc)
**12**	T4N2M0	33	70.0 Gy (22.6 cc)				59.4 Gy (486.1 cc)	59.4 Gy (410.9 cc)
**13**	T1N1M0	33	70.0 Gy (32.8 cc)				54.0 Gy (121.6 cc)	54.0 Gy (120.2 cc)
**14**	T1N1M0	33	70.0 Gy (49.4 cc)				59.4 Gy (327.5 cc)	54.0 Gy (242.5 cc)
**15**	T2N2M0	33	70.0 Gy (149.7 cc)				59.4 Gy (273.4 cc)	59.4 Gy (248.4 cc)
**16**	T2bN2M0	33	70.0 Gy (95.8 cc)	70.0 Gy (3.6 cc)			59.4 Gy (405.8 cc)	59.4 Gy (275.5 cc)
**17**	T4N3bM0	33	70.0 Gy (51.3 cc)	70.0 Gy (75.8 cc)			59.4 Gy (211.6 cc)	59.4 Gy (132.9 cc)
**18**	T1N2M0	33	70.0 Gy (90.3 cc)	70.0 Gy (13.9 cc)	70.0 Gy (5.8 cc)		59.4 Gy (175.5 cc)	59.4 Gy (177.8 cc)
**19**	T2aN2M0	33	70.0 Gy (8.5 cc)	70.0 Gy (7.9 cc)	70.0 Gy (77.5 cc)		59.4 Gy (225.5 cc)	59.4 Gy (329.5 cc)
**20**	T4N2M0	33	70.0 Gy (96.1 cc)				59.4 Gy (174.9 cc)	59.4 Gy (194.7 cc)

Table *S2* - Tolerance dose criteria for PTVs and OAR.

**Structures Name**	**Tolerance criteria**
PTVs	$D_{98\%} \geq D_{p,95\%}$
Spinal cord (SPNLCORD)	$D_{max} \leq 45Gy$
Brainstem	$D_{max} \leq 54Gy$
Chiasm	$D_{max} \leq 55Gy$
Left optical Nerve (OPTNRVL)	$D_{max} \leq 55Gy$
Right optical Nerve (OPTNRVR)	$D_{max} \leq 55Gy$
Left retina (RETINAL)	$D_{max} \leq 45Gy$
Right retina (RETINAR)	$D_{max} \leq 45Gy$
Left lens (LENSL)	$D_{max} \leq 6Gy$
Right lens (LENSR)	$D_{max} \leq 6Gy$
Left parotid (PAROTIDL)	$D_{mean} \leq 26Gy$
Right parotid (PAROTIDR)	$D_{mean} \leq 26Gy$
Oral cavity (ORALCAV)	$D_{mean} \leq 35Gy$
Oesophagus	$D_{mean} \leq 40Gy$
Larynx	$D_{mean} \leq 45Gy$
Left temporal mandibular junction (TMJL)	$D_{max} \leq 66Gy$
Right temporal mandibular junction (TMJR)	$D_{max} \leq 66Gy$
Mandible	$D_{max} \leq 66Gy$
Left ear canal (EARL)	$D_{mean} \leq 45Gy$
Right ear canal (EARR)	$D_{mean} \leq 45Gy$
Brain	$D_{max} \leq 54Gy$
Pituitary Gland (PITUITARY)	$D_{max} \leq 60Gy$
Thyroid	$D_{mean} \leq 27.5Gy$
Left lung (LUNGL)	$D_{mean} \leq 5Gy$
Right lung (LUNGR)	$D_{mean} \leq 5Gy$

D_p – Prescribed dose, D_{max} – maximum dose, D_{mean} – mean dose

Table S3 – Dose statistics of plans A and B of all patients calculated according the dose tolerance criteria defined in Table S2.

	**Patient #1**		**Patient #2**		**Patient #3**		**Patient #4**		**Patient #5**		**Patient #6**		**Patient #7**		**Patient #8**		**Patient #9**		**Patient #10**	
	**Plan A**	**Plan B**	**Plan A**	**Plan B**	**Plan A**	**Plan B**	**Plan A**	**Plan B**	**Plan A**	**Plan B**	**Plan A**	**Plan B**	**Plan A**	**Plan B**	**Plan A**	**Plan B**	**Plan A**	**Plan B**	**Plan A**	**Plan B**
**PTV70_T1**	67.7	67.8	65.7	66.8	65.3	67.1	67.4	67.8	67.5	67.9	66.3	66.5	68.1	68.7	67.2	67.3	68.4	68.3	67.7	68.1
**GTV70_N1**	67.0	67.1	65.3	67.4			67.1	67.3	67.1	65.1	67.7	67.7	66.1	66.1	67.5	67.9	67.6	68.2		
**GTV70_N2**	67.6	67.7	66.4	67.0									65.5	66.9	65.8	64.9				
**GTV70_N3**			64.3	66.7																
**PTV59_N1**	57.8	58.1	57.0	57.8	57.1	57.5	57.4	57.5	57.0	56.5	57.6	58.0	56.9	58.1	57.0	57.4	58.1	58.0	57.3	57.5
**PTV59_N2**	57.3	57.9	56.9	58.0	56.9	58.1							56.6	57.9	56.2	56.7	57.9	58.0	57.5	57.3
**PTV54_N1**																				
**PTV54_N2**							52.5	52.5	52.0	51.4	52.4	52.2								
**SPNLCORD**	44.1	44.0	47.4	45.1	46.2	44.0	44.7	44.4	44.6	45.0	45.6	45.4	47.6	42.5	48.3	45.1	43.3	44.0	45.7	44.9
**BRAINSTEM**	52.5	52.9	54.1	51.1	54.0	51.6	39.9	40.5	52.2	55.4	51.5	51.7	53.5	46.7	53.5	50.8	48.0	48.0	52.7	52.4
**LENSR**	1.6	1.8	1.0	2.4	18.8	10.2	0.6	0.6	0.3	0.1	0.9	0.8	1.0	1.2	7.0	3.9	1.1	1.5	1.4	1.5
**LENSL**	1.2	1.6	1.7	2.1	32.2	13.0	0.5	0.2	0.3	0.0	1.1	0.9	0.5	0.3	8.0	3.1	0.9	1.0	1.4	1.3
**ONR**	4.9	4.6	4.4	3.9	55.0	52.7			2.4	2.1			2.8	2.5	11.3	9.6	3.1	3.2	3.6	3.4
**ONL**	5.8	5.5	2.5	2.6	55.0	52.7			1.8	1.4			2.8	2.6	8.2	8.1	3.4	3.3	3.9	3.4
**RETINAR**	4.0	4.6	7.3	11.7	50.7	37.8	2.3	2.5	1.6	1.2	2.4	2.2	3.9	4.3	11.8	11.6	2.2	2.6	6.3	5.9
**RETINAL**	5.0	6.7	11.9	12.2	46.0	28.1	1.6	1.1	1.5	0.5	2.6	2.3	1.6	1.3	26.6	17.2	2.2	2.5	14.5	7.3
**CHIASM**	6.2	5.7			55.0	52.8			2.9	3.0	5.0	5.1	2.0	1.9	11.3	9.6	5.4	5.6	4.1	4.0
**TMJR**	51.6	54.6			66.0	66.0			66.0	70.6	66.0	66.0			59.0	59.0	59.3	60.3	46.1	54.2
**TMJL**	66.0	66.0			63.2	64.4			57.2	56.2	56.3	56.0			65.7	64.9	62.0	60.5	54.3	53.5
**EARR**					31.8	34.7			21.0	26.8	35.4	37.2	35.0	32.5	13.0	23.8	39.4	38.8	18.9	24.6
**EARL**					28.9	28.9			19.7	18.3	28.6	30.8	34.6	33.0	44.4	38.0	43.7	41.8	16.3	18.1
**MANDIBLE**	66.0	66.2	65.6	66.5	60.4	60.0	60.7	61.0	63.0	61.4	67.2	67.3	61.2	61.7	69.8	69.1	61.3	61.0	62.3	62.0
**BRAIN**	62.6	61.9			75.9	75.6	65.2	63.8	58.4	61.8	72.4	72.6	53.3	54.1	66.3	68.5	61.3	60.3	48.9	52.6
**PITUITARY**	28.9	25.4			55.3	58.5			6.7	7.3	21.6	22.0			35.2	30.2	13.9	13.6	6.1	5.7
**LUNGR**	0.9	0.8	4.6	4.9	5.0	4.3	2.3	2.7	1.7	1.6	1.0	1.2			2.0	2.3	4.1	4.9	4.9	4.9
**LUNGL**	1.6	1.9	9.3	5.0	5.9	5.2	2.2	1.7	1.1	2.0	1.3	1.3			2.8	2.3	5.0	5.0	4.8	4.8
**THYROID**	35.1	32.1	60.2	54.0	50.6	47.8	35.0	31.5	27.8	46.6	38.1	30.9	47.9	28.0	51.0	34.9	27.9	27.9	28.8	28.0
**ORALCAV**	35.1	35.2			32.5	38.4	35.2	35.2	35.2	34.8	35.1	35.0	40.2	35.2	33.0	35.2	35.4	35.2	35.1	35.1
**OESOPHAGUS**	21.2	22.5	29.6	31.0	37.2	30.1					14.6	16.9			35.3	17.7	34.4	34.3	24.2	23.8
**LARYNX**	34.8	34.9	47.0	35.8	45.5	38.6			35.0	41.6	34.9	34.4	45.4	35.6	41.0	35.0	34.1	35.2	35.2	35.1
**PAROTIDR**	25.9	25.9	43.1	26.0	48.2	39.0	25.9	25.9	26.0	42.6	26.0	26.0	36.2	26.1	40.1	26.3	26.1	26.1	26.3	26.3
**PAROTIDL**	26.0	26.0	48.2	25.9	44.2	39.3	26.0	26.0	25.9	39.7	25.9	25.9	37.6	26.0	31.6	26.2	26.1	26.2	25.8	25.8

Table S3 (cont)

	Patient #11		Patient #12		Patient #13		Patient #14		Patient #15		Patient #16		Patient #17		Patient #18		Patient #19		Patient #20	
	Plan A	Plan B	Plan A	Plan B	Plan A	Plan B	Plan A	Plan B	Plan A	Plan B	Plan A	Plan B	Plan A	Plan B	Plan A	Plan B	Plan A	Plan B	Plan A	Plan B
**PTV70_T1**	66.9	67.2	67.8	66.8	68.0	68.3	67.1	66.7	67.2	67.4	67.7	67.7	66.2	66.2	60.8	60.2	65.8	66.2	64.5	67.6
**GTV70_N1**											66.2	65.8	68.4	68.9	64.4	64.9	64.9	65.2		
**GTV70_N2**															63.7	64.4	68.0	67.8		
**GTV70_N3**																				
**PTV59_N1**	58.7	57.9	57.3	56.4			57.8	56.9	57.0	57.0	57.8	57.9	58.4	58.6	56.6	56.7	56.7	56.6	57.2	56.4
**PTV59_N2**	56.7	56.0	57.7	56.7					56.3	57.1	57.4	57.3	57.2	56.4	57.0	57.2	57.0	57.0	56.5	56.9
**PTV54_N1**					52.1	51.9														
**PTV54_N2**					51.9	51.7	52.5	51.4												
**SPNLCORD**	46.0	44.6	46.4	45.5	44.5	45.4	44.9	46.3	46.5	48.8	44.1	44.0	45.9	45.2	45.8	44.0	44.5	44.4	46.0	45.1
**BRAINSTEM**	52.9	55.3	52.2	52.6	51.3	53.3	53.3	55.1	53.3	53.8	51.8	52.1	53.2	52.9	58.5	53.4	53.0	53.3	57.1	60.6
**LENSR**	3.7	6.9	6.1	6.3	6.3	2.1	0.8	1.1	1.5	1.1	7.1	7.3	0.5	0.5	37.8	19.3	1.7	1.1	1.4	1.5
**LENSL**	2.0	1.5	5.6	6.3	2.4	3.5	0.5	0.4	1.5	1.4	6.4	6.8	1.4	1.1	25.1	24.3	1.2	1.3	1.2	1.5
**ONR**	15.5	17.9	52.3	53.9	2.0	2.8	3.8	4.7	6.6	6.1	23.8	28.6			46.5	45.2	4.9	6.4	25.5	23.6
**ONL**	15.6	15.8	30.3	30.9	4.1	4.7	4.4	4.0	6.4	6.7	19.3	18.4			55.0	55.0	3.0	4.1	38.9	40.0
**RETINAR**	8.3	14.3	18.5	27.7	8.9	8.1	2.8	4.0	7.3	5.1	36.0	33.6			44.5	45.0	8.2	10.2	6.8	8.2
**RETINAL**	4.2	2.9	12.8	12.5	6.7	8.0	2.7	2.3	7.0	6.3	22.3	24.7			45.0	45.0	8.4	10.7	13.1	9.2
**CHIASM**	17.7	18.6	52.3	53.9	3.3	3.4	3.4	3.3	18.5	21.4	23.8	28.6			55.0	55.0	2.8	2.9	43.7	50.4
**TMJR**	66.0	73.0	52.6	57.4			53.7	57.2			64.2	65.7	54.1	45.3					60.3	60.2
**TMJL**	66.0	68.8	53.2	48.1			58.4	59.9			62.7	64.2	66.0	66.0					65.0	64.4
**EARR**	44.6	44.7	45.0	45.0	27.7	20.7	28.4	22.6	26.8	26.7	30.3	29.4	27.1	20.3	42.6	39.5	25.4	26.8	46.5	36.2
**EARL**	40.2	41.8	36.3	21.1	22.5	23.5	36.0	31.6	34.9	34.7	34.9	35.6	30.8	32.4	26.2	23.8	17.5	28.7	43.1	35.9
**MANDIBLE**	70.6	75.0	63.4	60.9	61.7	62.5	63.4	61.3	66.5	66.7	63.7	64.3	63.9	66.8	58.8	57.7	62.7	63.6	61.1	60.5
**BRAIN**	74.4	75.1	76.2	75.7	62.2	63.4	60.9	61.0	73.0	71.7	63.4	62.4	66.0	64.8			58.8	60.3	74.9	75.9
**PITUITARY**	50.7	54.1	62.8	65.0	4.9	5.1	7.5	7.3	54.3	53.5	31.6	37.3					6.9	7.2	69.3	74.6
**LUNGR**	2.7	2.6	1.7	1.6			3.3	2.8	0.5	0.2	2.5	2.6	2.5	2.7			1.8	2.0	0.8	0.7
**LUNGL**	1.9	1.6	0.7	0.8			4.6	3.7	1.2	1.3	3.7	3.6	3.1	3.2			2.8	2.6	0.4	0.5
**THYROID**	57.4	59.0	51.9	50.1	43.5	42.9	27.5	41.0	55.8	45.2	33.9	33.5	29.4	29.8	56.6	57.0	53.9	55.2	50.4	42.3
**ORALCAV**	45.3	45.2	35.0	45.0			35.4	33.3	35.0	35.3	35.3	35.4	35.3	35.2	37.0	35.8	43.4	42.3	30.6	35.0
**OESOPHAGUS**	33.8	33.8	12.0	10.8			14.9	20.2	34.1	33.7	25.0	25.7	34.8	33.8	42.9	39.6	20.4	19.9	31.3	16.6
**LARYNX**	35.4	43.4	35.3	35.2			35.0	44.4	44.8	35.2	35.0	35.0	35.3	35.3	48.0	46.6			46.6	35.2
**PAROTIDR**	35.4	51.5	25.7	27.9	26.1	26.0	26.1	40.6	26.0	26.0	26.1	26.1	26.1	26.0	40.2	41.3	31.9	33.2	44.9	26.3
**PAROTIDL**	27.9	35.7	26.1	29.0	26.1	26.0	26.1	43.1	30.3	26.2	25.9	25.9	26.2	28.0	42.3	41.6	34.5	34.0	46.6	26.2

Table S4 – SPIDERplan structures scores of plans A and B calculated according equations 2 and 3.

	**Patient #1**		**Patient #2**		**Patient #3**		**Patient #4**		**Patient #5**		**Patient #6**		**Patient #7**		**Patient #8**		**Patient #9**		**Patient #10**	
	**Plan A**	**Plan B**	**Plan A**	**Plan B**	**Plan A**	**Plan B**	**Plan A**	**Plan B**	**Plan A**	**Plan B**	**Plan A**	**Plan B**	**Plan A**	**Plan B**	**Plan A**	**Plan B**	**Plan A**	**Plan B**	**Plan A**	**Plan B**
**PTV70_T1**	0.982	0.981	1.012	0.995	1.019	0.990	0.986	0.980	0.985	0.978	1.002	0.999	0.975	0.967	0.989	0.987	0.972	0.973	0.982	0.977
**GTV70_N1**	0.992	0.991	1.019	0.986			0.991	0.987	0.990	1.021	0.982	0.982	1.006	1.005	0.984	0.978	0.982	0.975		
**GTV70_N2**	0.983	0.982	1.002	0.992									1.015	0.994	1.010	1.025				
**GTV70_N3**			1.034	0.996																
**PTV59_N1**	0.975	0.971	0.990	0.977	0.988	0.982	0.983	0.981	0.991	0.998	0.979	0.974	0.991	0.971	0.990	0.983	0.972	0.973	0.985	0.981
**PTV59_N2**	0.985	0.974	0.992	0.972	0.992	0.972							0.997	0.975	1.004	0.996	0.974	0.973	0.982	0.986
**PTV54_N1**																				
**PTV54_N2**							0.978	0.976	0.987	0.999	0.979	0.983								
**SPNLCORD**	0.980	0.979	1.052	1.002	1.028	0.979	0.993	0.986	0.991	1.001	1.014	1.009	1.057	0.945	1.074	1.003	0.962	0.978	1.016	0.998
**BRAINSTEM**	0.971	0.979	1.002	0.946	0.999	0.955	0.739	0.750	0.966	1.025	0.953	0.958	0.992	0.865	0.991	0.940	0.889	0.888	0.975	0.970
**LENSR**	0.265	0.300	0.172	0.405	3.130	1.694	0.095	0.096	0.046	0.021	0.149	0.139	0.162	0.200	1.163	0.648	0.190	0.242	0.226	0.244
**LENSL**	0.203	0.260	0.281	0.344	5.365	2.173	0.082	0.039	0.046	0.007	0.182	0.149	0.077	0.055	1.333	0.511	0.145	0.161	0.233	0.216
**ONR**	0.090	0.083	0.080	0.071	1.000	0.959			0.043	0.039			0.050	0.045	0.206	0.175	0.057	0.058	0.065	0.062
**ONL**	0.106	0.101	0.045	0.047	1.000	0.959			0.033	0.025			0.050	0.048	0.150	0.148	0.061	0.060	0.072	0.061
**RETINAR**	0.089	0.101	0.163	0.259	1.128	0.840	0.052	0.055	0.035	0.027	0.053	0.050	0.087	0.096	0.262	0.258	0.049	0.057	0.140	0.132
**RETINAL**	0.110	0.148	0.264	0.271	1.023	0.624	0.036	0.025	0.033	0.012	0.059	0.051	0.035	0.030	0.591	0.383	0.048	0.056	0.322	0.163
**CHIASM**	0.112	0.104			1.000	0.959			0.052	0.055	0.092	0.093	0.037	0.035	0.205	0.175	0.097	0.101	0.075	0.073
**TMJR**	0.781	0.827			1.000	1.000			1.000	1.070	1.000	1.000			0.894	0.894	0.899	0.913	0.699	0.821
**TMJL**	1.000	1.000			0.958	0.975			0.866	0.852	0.853	0.849			0.995	0.984	0.939	0.916	0.822	0.811
**EARR**					0.706	0.771			0.468	0.597	0.787	0.827	0.778	0.722	0.289	0.528	0.875	0.863	0.419	0.546
**EARL**					0.641	0.642			0.439	0.407	0.635	0.685	0.768	0.734	0.986	0.845	0.971	0.929	0.363	0.402
**MANDIBLE**	1.000	1.003	0.995	1.008	0.914	0.909	0.920	0.924	0.955	0.930	1.018	1.019	0.928	0.936	1.058	1.047	0.929	0.924	0.944	0.940
**BRAIN**	1.160	1.146			1.405	1.401	1.207	1.182	1.081	1.144	1.340	1.345	0.987	1.002	1.228	1.269	1.135	1.117	0.906	0.973
**PITUITARY**	0.482	0.423			0.922	0.974			0.112	0.121	0.360	0.367			0.587	0.503	0.231	0.226	0.102	0.094
**LUNGR**	0.171	0.163	0.930	0.975	0.994	0.863	0.459	0.548	0.337	0.319	0.198	0.250			0.401	0.455	0.810	0.973	0.972	0.975
**LUNGL**	0.328	0.373	1.865	0.990	1.183	1.044	0.430	0.337	0.230	0.392	0.252	0.255			0.551	0.466	0.994	0.992	0.966	0.968
**THYROID**	1.275	1.166	2.189	1.964	1.842	1.738	1.271	1.146	1.009	1.696	1.386	1.123	1.743	1.017	1.856	1.270	1.013	1.013	1.046	1.018
**ORALCAV**	1.004	1.007			0.928	1.098	1.005	1.006	1.005	0.994	1.002	1.001	1.149	1.005	0.942	1.007	1.012	1.006	1.003	1.003
**OESOPHAGUS**	0.530	0.562	0.739	0.774	0.929	0.751					0.366	0.423			0.883	0.442	0.861	0.858	0.605	0.596
**LARYNX**	0.773	0.775	1.045	0.795	1.012	0.857			0.778	0.925	0.777	0.764	1.008	0.792	0.911	0.778	0.757	0.781	0.783	0.781
**PAROTIDR**	0.996	0.996	1.656	0.998	1.856	1.501	0.996	0.996	1.000	1.637	0.999	0.999	1.393	1.002	1.543	1.012	1.004	1.002	1.011	1.012
**PAROTIDL**	1.000	0.999	1.855	0.997	1.699	1.511	1.000	0.999	0.996	1.528	0.995	0.998	1.445	0.999	1.216	1.007	1.006	1.006	0.993	0.992

Table S4 (cont)

	Patient #11		Patient #12		Patient #13		Patient #14		Patient #15		Patient #16		Patient #17		Patient #18		Patient #19		Patient #20	
	**Plan A**	**Plan B**	**Plan A**	**Plan B**	**Plan A**	**Plan B**	**Plan A**	**Plan B**	**Plan A**	**Plan B**	**Plan A**	**Plan B**	**Plan A**	**Plan B**	**Plan A**	**Plan B**	**Plan A**	**Plan B**	**Plan A**	**Plan B**
**PTV70_T1**	0.994	0.988	0.981	0.996	0.977	0.973	0.990	0.997	0.989	0.986	0.982	0.981	1.005	1.003	1.092	1.103	1.009	1.003	1.030	0.983
**GTV70_N1**											1.004	1.010	0.972	0.965	1.033	1.024	1.024	1.020		
**GTV70_N2**															1.044	1.031	0.977	0.980		
**GTV70_N3**																				
**PTV59_N1**	0.961	0.974	0.985	1.001			0.977	0.991	0.991	0.989	0.977	0.974	0.966	0.963	0.996	0.995	0.996	0.997	0.987	1.001
**PTV59_N2**	0.995	1.008	0.979	0.995					1.003	0.989	0.983	0.984	0.987	1.000	0.991	0.987	0.989	0.990	0.998	0.992
**PTV54_N1**					0.984	0.988														
**PTV54_N2**					0.989	0.991	0.977	0.998												
**SPNLCORD**	1.021	0.991	1.032	1.011	0.989	1.010	0.998	1.029	1.032	1.084	0.980	0.978	1.021	1.004	1.019	0.979	0.989	0.988	1.023	1.003
**BRAINSTEM**	0.979	1.024	0.967	0.975	0.950	0.987	0.987	1.020	0.986	0.997	0.958	0.965	0.984	0.980	1.084	0.990	0.982	0.987	1.057	1.123
**LENSR**	0.612	1.148	1.018	1.051	1.044	0.354	0.127	0.176	0.256	0.191	1.190	1.221	0.080	0.087	6.302	3.217	0.279	0.188	0.238	0.258
**LENSL**	0.330	0.250	0.929	1.056	0.398	0.584	0.083	0.072	0.252	0.238	1.066	1.138	0.236	0.179	4.180	4.053	0.205	0.212	0.198	0.256
**ONR**	0.282	0.325	0.950	0.979	0.036	0.050	0.070	0.085	0.120	0.110	0.433	0.520			0.845	0.822	0.090	0.117	0.464	0.428
**ONL**	0.283	0.288	0.550	0.563	0.075	0.085	0.079	0.073	0.116	0.123	0.351	0.335			1.000	1.000	0.055	0.075	0.707	0.727
**RETINAR**	0.185	0.317	0.412	0.615	0.198	0.179	0.062	0.088	0.163	0.113	0.801	0.747			0.990	1.000	0.183	0.227	0.151	0.183
**RETINAL**	0.094	0.065	0.283	0.279	0.149	0.178	0.059	0.050	0.155	0.140	0.495	0.549			1.000	1.000	0.186	0.238	0.292	0.205
**CHIASM**	0.322	0.339	0.950	0.979	0.060	0.063	0.062	0.060	0.337	0.389	0.433	0.520			1.000	1.000	0.050	0.052	0.794	0.916
**TMJR**	1.000	1.107	0.798	0.870			0.813	0.866			0.973	0.995	0.819	0.686					0.913	0.913
**TMJL**	1.000	1.042	0.806	0.728			0.885	0.908			0.951	0.973	1.000	1.000					0.984	0.976
**EARR**	0.992	0.993	1.000	1.000	0.615	0.460	0.632	0.502	0.595	0.594	0.673	0.654	0.603	0.451	0.946	0.877	0.565	0.595	1.034	0.804
**EARL**	0.894	0.929	0.806	0.469	0.501	0.522	0.800	0.703	0.777	0.771	0.776	0.790	0.684	0.720	0.582	0.528	0.390	0.639	0.957	0.799
**MANDIBLE**	1.070	1.137	0.961	0.922	0.935	0.947	0.960	0.928	1.007	1.011	0.965	0.975	0.968	1.012	0.891	0.874	0.950	0.963	0.926	0.917
**BRAIN**	1.378	1.390	1.411	1.402	1.152	1.173	1.127	1.130	1.352	1.328	1.174	1.155	1.221	1.200			1.089	1.116	1.386	1.405
**PITUITARY**	0.844	0.901	1.046	1.083	0.082	0.086	0.124	0.121	0.905	0.891	0.527	0.622					0.115	0.120	1.155	1.243
**LUNGR**	0.540	0.520	0.336	0.321			0.653	0.550	0.105	0.047	0.495	0.521	0.500	0.532			0.363	0.395	0.157	0.139
**LUNGL**	0.374	0.318	0.131	0.157			0.915	0.747	0.249	0.253	0.731	0.727	0.619	0.636			0.554	0.511	0.070	0.109
**THYROID**	2.088	2.146	1.886	1.821	1.582	1.560	1.001	1.492	2.030	1.643	1.231	1.219	1.070	1.085	2.057	2.073	1.960	2.007	1.833	1.539
**ORALCAV**	1.294	1.292	1.001	1.286			1.011	0.951	1.000	1.009	1.009	1.012	1.008	1.006	1.056	1.022	1.239	1.207	0.875	1.000
**OESOPHAGUS**	0.845	0.845	0.299	0.270			0.373	0.504	0.853	0.843	0.626	0.642	0.871	0.846	1.072	0.990	0.509	0.498	0.781	0.416
**LARYNX**	0.786	0.965	0.784	0.782			0.777	0.986	0.995	0.783	0.778	0.778	0.785	0.784	1.066	1.036			1.035	0.781
**PAROTIDR**	1.361	1.980	0.990	1.073	1.004	0.999	1.003	1.563	1.001	0.999	1.003	1.002	1.003	0.999	1.547	1.590	1.226	1.278	1.725	1.011
**PAROTIDL**	1.072	1.372	1.002	1.116	1.002	1.000	1.003	1.656	1.164	1.006	0.997	0.996	1.009	1.077	1.626	1.600	1.328	1.308	1.792	1.008

Patient DVH example

Patient #3

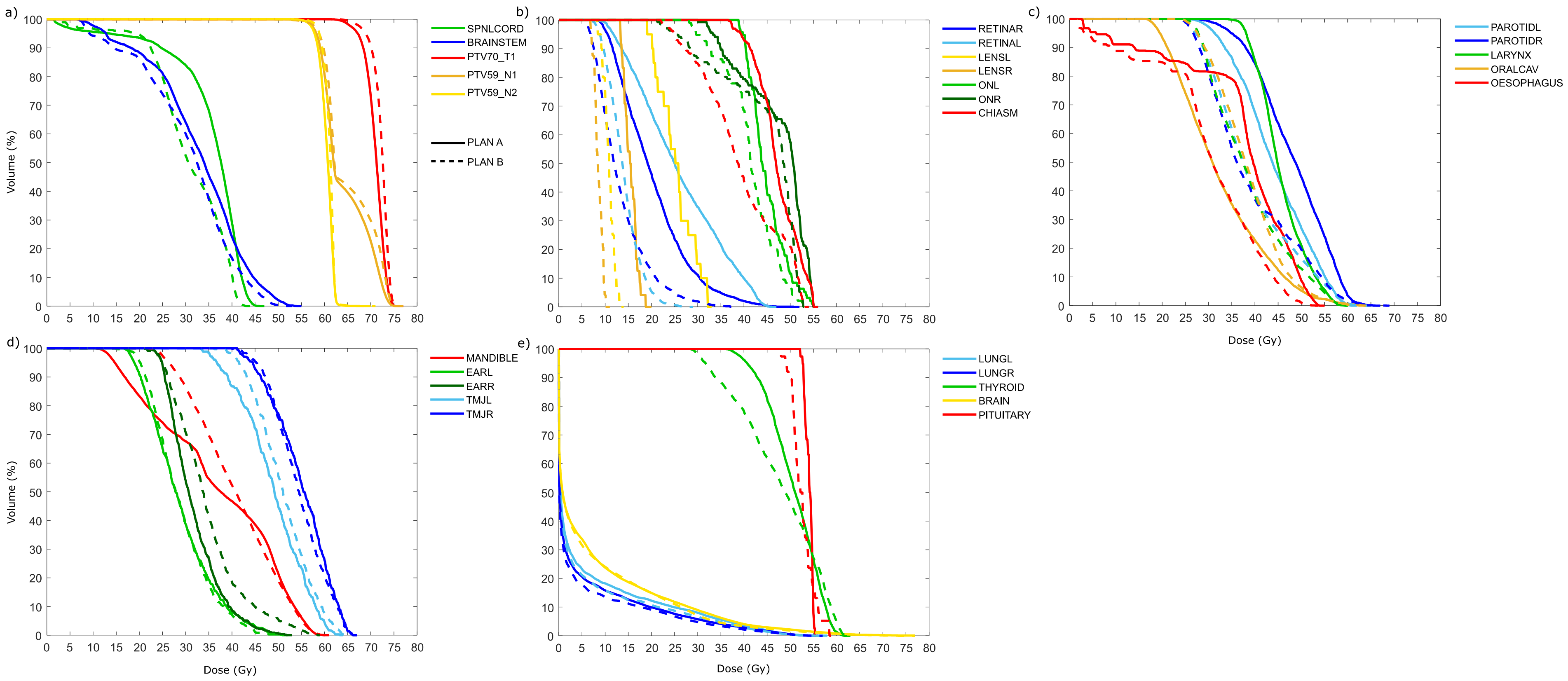

Figure S1- DVHs of plans A and B of patient #3 for a) PTVs, spinal cord and brainstem; b) retinas, lenses, optical nerves and chiasm; c) parotids, larynx, oral cavity and oesophagus; d) mandible, ears, temporal mandibular junction and e) lungs, thyroid, brain and pituitary.
